# Current understanding of cancer stem cells: Immune evasion and targeted immunotherapy in gastrointestinal malignancies

**DOI:** 10.3389/fonc.2023.1114621

**Published:** 2023-02-23

**Authors:** Junyi An, Xiaohua Hu, Feng Liu

**Affiliations:** Department of Oncology, Shanghai Ninth People’s Hospital, Shanghai Jiao Tong University School of Medicine, Shanghai, China

**Keywords:** cancer stem cells, immunotherapy, gastrointestinal malignancies, anti-tumor therapy, immune evasion

## Abstract

As a relatively rare population of cancer cells existing in the tumor microenvironment, cancer stem cells (CSCs) possess properties of immune privilege to evade the attack of immune system, regulated by the microenvironment of CSCs, the so-called CSCs niche. The bidirectional interaction of CSCs with tumor microenvironment (TME) components favors an immunosuppressive shelter for CSCs’ survival and maintenance. Gastrointestinal cancer stem cells (GCSCs) are broadly regarded to be intimately involved in tumor initiation, progression, metastasis and recurrence, with elevated tumor resistance to conventional therapies, which pose a major hindrance to the clinical efficacy for treated patients with gastrointestinal malignancies. Thus, a multitude of efforts have been made to combat and eradicate GCSCs within the tumor mass. Among diverse methods of targeting CSCs in gastrointestinal malignancies, immunotherapy represents a promising strategy. And the better understanding of GCSCs immunomodulation and immunoresistance mechanisms is beneficial to guide and design novel GCSCs-specific immunotherapies with enhanced immune response and clinical efficacy. In this review, we have gathered available and updated information to present an overview of the immunoevasion features harbored by cancer stem cells, and we focus on the description of immune escape strategies utilized by CSCs and microenvironmental regulations underlying CSCs immuno-suppression in the context of gastrointestinal malignancies. Importantly, this review offers deep insights into recent advances of CSC-targeting immunotherapeutic approaches in gastrointestinal cancers.

## 1 Introduction

Gastrointestinal malignancies mainly including colorectal cancer, gastric cancer, pancreatic cancer, liver cancer and esophageal cancer, are still serious public health problems with worldwide concern. According to GLOBOCAN 2020 report, colorectal cancer (CRC) ranks as the second greatest cause of cancer-related mortality globally (1). In the United States, CRC has been estimated as the second deadliest cancer type among men and women combined and it ranks third for incidence in both men and women (2). Despite different therapeutic tools like surgery, radiotherapy, and chemotherapy, a certain number of patients still face unremarkable and unfavorable clinical outcomes owing to therapeutic resistance, tumor metastasis and recurrence. Alternatively, immunotherapies such as cancer vaccines, immune checkpoint inhibitors and adoptive cell transfer can stimulate anti-tumor immune responses to acquire long-term efficacy. Therefore, immunotherapy has become an optimal treatment modality for the favorable prognosis of patients with gastrointestinal malignancies.

**Figure 1:**
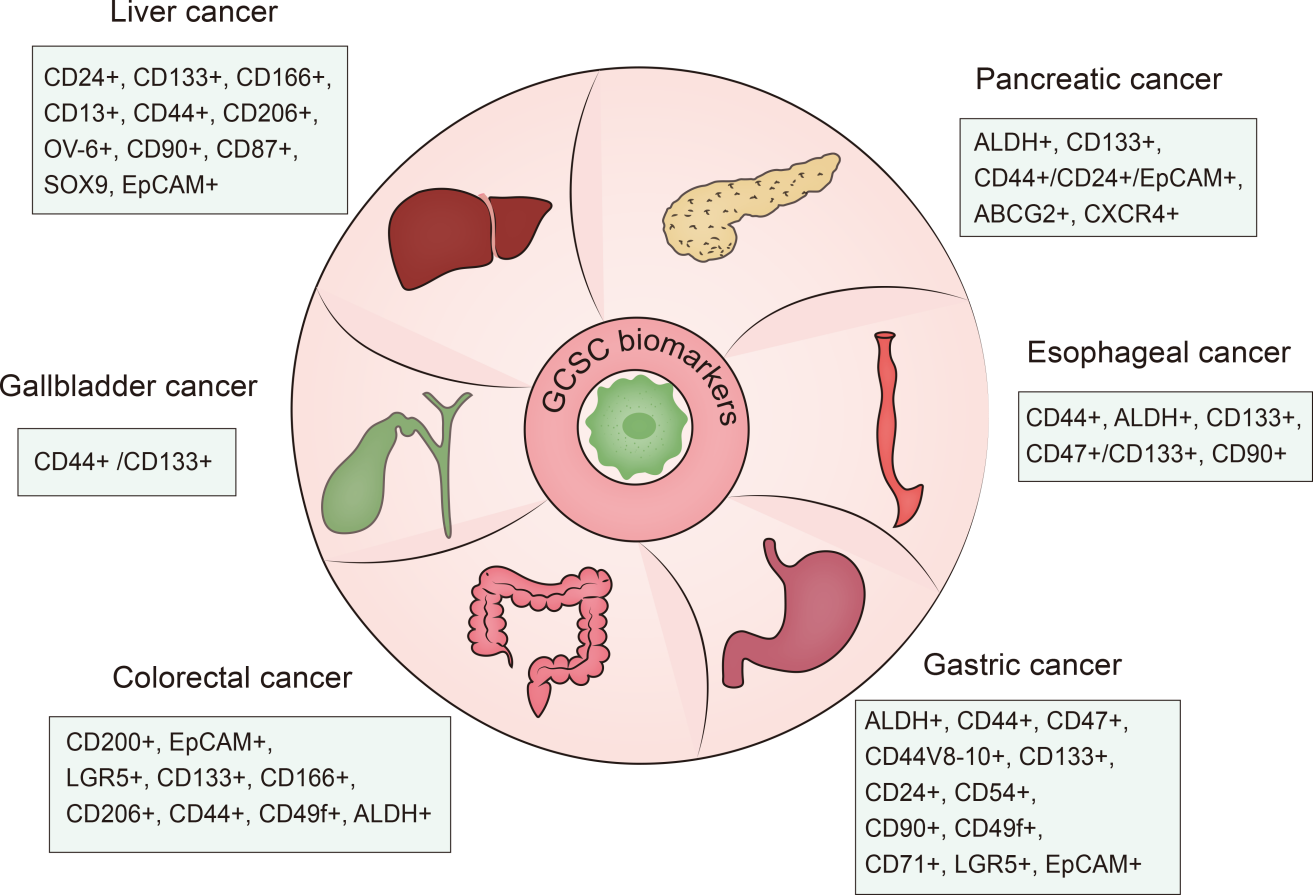
Summary of GCSC biomarkers. GCSC, gastrointestinal cancer stem cell.

Cancer stem cells also referred to as tumor initiating cells (TICs) or cancer progenitor cells, are a small group of unlimitedly proliferative tumor cells endowed with tumorigenicity along with stem cell-like traits like self-renewal, proliferation, quiescence and differentiation. It was reported that CSCs were first identified from acute myeloid leukemia (AML) by Bonnet and Dick in the late 1990s (3). Subsequently, researchers further found the existence of CSCs in multiple solid tumor types including cancers of the colorectal (4, 5), pancreas (6), liver (7, 8), gastric (9), brain (10, 11), breast (12–15), prostate (16), lung (17), bladder (18) and melanoma (19). Notably, cancer stem cells in gastrointestinal cancers were first detected in 2007 in colorectal cancer (5). Different biomarkers of gastrointestinal cancer stem cell can be used for identification and antitumor therapies (20–22) (**Figure 1**). GCSCs are located in a specialized environment called “niche”, which can support and maintain the properties of immune privilege harbored by GCSCs. The properties and the suppressive environment enable GCSCs to be resistant to conventional treatment strategies. According to the accumulated evidence, GCSCs possess therapeutic resistance through several mechanisms including the maintenance of senescence, increased DNA repair capacity and redox capacity, drug efflux, and epithelial-mesenchymal transition (EMT) (23, 24). Therefore, targeting GCSCs is a promising method.

GCSCs are capable of evading immune system impairment through adopting a series of smart strategies and interacting with the tumor microenvironment. In the context of gastrointestinal cancers, enough consideration has been given to elucidating the relevant strategies CSCs adopted to evade immune attacks, which contribute to therapy insensitivity and drug resistance. Additionally, this review offers a current understanding of GCSCs and their niche with a special emphasis on bidirectional crosstalk. To precisely target GCSCs, this review highlights various immune-targeted therapies against gastrointestinal cancers for the purpose of exploring more effective GCSCs-specific targeted therapeutic tools.

## 2 Immune escape mechanisms of GCSCs

**Figure 2:**
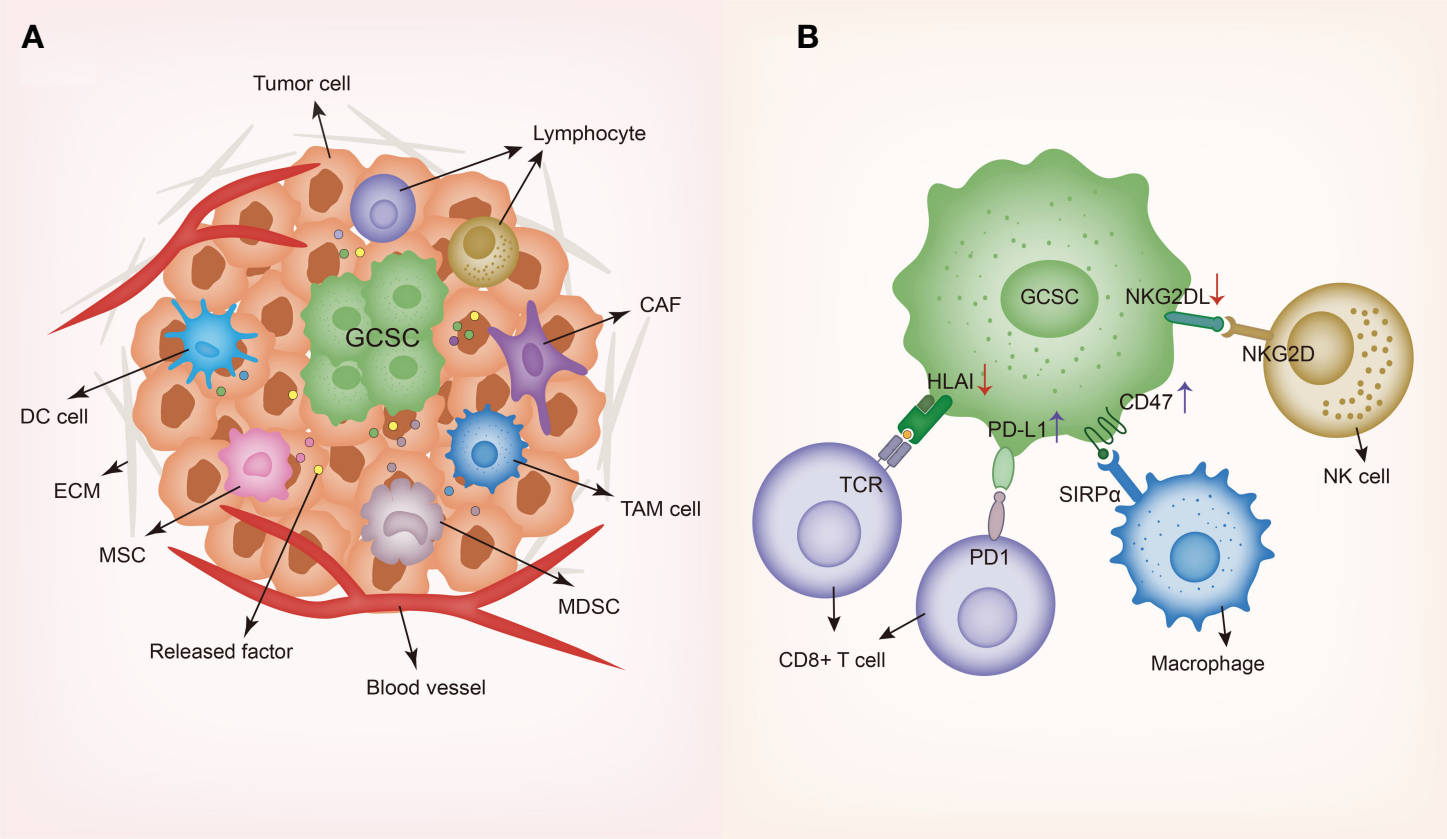
Schematic representing gastrointestinal cancer stem cells and their niche. **(A)** GCSC niche contains diverse cell types such as dendritic cells (DCs), tumor-associated macrophages (TAMs), cancer-associated fibroblasts (CAFs), myeloid-derived suppressor cells (MDSCs), cancer-associated mesenchymal stem cells (MSCs) and lymphocytes, along with various secreted factors such as cytokines and chemokines. In order to create and maintain an immunosuppressive environment, GCSCs recruit different cells to the niche and modulate the behaviors of these cells through producing a wide variety of soluble factors. **(B)** Immune evasion mechanisms of GCSC including the downregulation of HLA and NKG2DL and the upregulation of PD-L1 and CD47. GCSC, gastrointestinal cancer stem cell; DC, dendritic cell; TAM, tumor-associated macrophage; CAF, cancer-associated fibroblast; MDSC, myeloid-derived suppressor cell; MSC, mesenchymal stem cell; ECM, extracellular matrix; HLA, human leukocyte antigen-A; SIRPα, signal regulatory protein alpha; TCR, T cell receptor; NKG2D, natural killer group 2D; NKG2DL, natural killer group 2D ligand.

Certain methods have been adopted by GCSCs to circumvent the attack of immune system, enhance their own survival and facilitate tumor initiation, about which we will discuss as the followings (**Figure 2**). GCSCs can not only downregulate relevant molecules expression to suppress T cells response and NK cells-mediated cytotoxicity, but also exhibit increased levels of immune checkpoint molecules expression to support immune evasion.

### 2.1 Reduced expression of MHC-I and natural killer ligands

A growing number of studies highlight that CSCs exhibit reduced expression of the components that participate in antigen processing and presentation, for instance, the major histocompatibility complex class I (MHC- I) molecules (HLA-A, B, C) and the antigen processing machinery (APM) molecules, suggesting CSCs ability to avoid the recognition and activation of CD8^+^ T lymphocytes. Such a phenomenon was observed in CSCs from glioblastoma (25), melanoma (26), lung cancer (27), head and neck squamous cell carcinoma (28, 29), and colorectal cancer (30, 31), rather than the non-CSCs counterparts. Importantly, CSCs with a decreased level of MHC-I expression are vulnerable to NK cells. For example, CRC CICs characteristic of lower MHC-I expression was found to show higher NK cell susceptibility due to high levels of NK-cell-activating receptor ligands (30).

Nevertheless, CSCs can also evade NK cells-mediated killing by tuning the expression of NK cell activating and inhibitory receptors (32). It was observed that STAT3 could suppress NK cell-mediated immunosurveillance through downregulating natural killer group 2, member D receptor ligands (NKG2DL) in HT29 colorectal cancer cell line, and STAT3 neutralization activated NK cells *via* the induction of MHC class I chain-related protein A (MICA), which is the recognition receptor of NK cells (33). In addition, one study showed NK cell-mediated cytotoxicity can be attenuated through upregulating carcinoembryonic antigen-related cell adhesion molecule 1 (CEACAM1) of EpCAM^high^ liver CSCs (34). Using anti-CEACAM1 antibody can inhibit EpCAM^high^ CSCs in hepatocellular carcinoma (HCC).

### 2.2 Elevated expression of immune checkpoint molecules

It has been found that PD-1 ligand (PD-L1), also known as B7H1 or CD274, is overexpressed on CSCs from CRC (35–37), head and neck squamous cell carcinoma (38), and gastric cancer (39), contributing to immune evasion of CSCs (40). EMT induced PD-L1 expressed on CSCs *via* EMT/β-catenin/STT3/PD-L1 signaling axis, and targeting EMT/β-catenin/STT3/PD-L1 axis may downregulate PD-L1 (40). In colorectal cancer, PD-L1 can trigger CSC-like characteristics and chemoresistance in CRC cells (41). PD-L1 overexpressed on CD133^+^ colorectal CSCs and EMT enable CSCs to invade and metastasize (37). Moreover, PD-L1 was found to be overexpressed on CD133^+^CD44^+^ colorectal CSCs, and it promoted CSCs expansion and self-renewal *via* PD-L1- activated HMGA1-dependent signaling pathways (36). In gastric cancer, B7-H1 expressed in Lgr5^+^ gastric CSCs can enhance CSC proliferation and tumor formation by binding with PD-1 on T cells (39). Besides, B7-H3 was reported to be expressed on ovarian cancer initiating cells (CICs), and applying B7-H3-specific monoclonal antibody 376.96 resulted in the reduction of CICs content *in vitro* (42).

Based on the existing evidence, the “don’t eat me” signal CD47 has been reported to exhibit elevated levels of CSCs from liver cancer (43, 44), pancreatic cancer (45), esophageal squamous cell carcinoma (ESCC) (46), lung cancer (47) and other cancer types. CD47 can inhibit the phagocytic activity of macrophages to cancer stem cells by interacting with signal regulatory protein α (SIRP-α) on phagocytic cells. In addition, Michele Cioffi et al. (45) demonstrated that anti-CD47 treatment induced pancreatic CSCs apoptosis apart from being phagocytized by macrophages. The upregulation of CD47 assists CSCs to escape destruction from the immune system. In ESCC, researchers found CD47^+^ CD133^+^ ESCC cells with CSC-like characteristics can be veritably eliminated after anti-CD47 treatment (46). It has been found that 4-methylumbelliferone (4Mu), the hyaluronan synthesis inhibitor, plays a role in promoting phagocytosis *via* downregulating CD47 expression on hepatic CSCs, and potentiating cytotoxic-specific T cell response against HCC induced by interleukin-12 (48). Additionally, suppression of CD47 preferentially expressed on liver TICs reduced HCC CSC-like properties including self-renewal and chemoresistance (43).

## 3 Microenvironmental regulations of GCSCs

As widely regarded, the tumor microenvironment CSCs reside in has been termed as “CSCs niche” and accumulated studies indicate that there exists complicated interplay between CSCs and the tumor microenvironment (TME), playing a pivotal role in tumor invasiveness and progression. Studies have uncovered that different microenvironmental regulation factors collaborate with GCSCs to sustain their immunosuppression properties. Especially, GCSCs interact with different cells in the TME composed of dendritic cells (DCs), tumor-associated macrophages (TAMs), cancer-associated fibroblasts (CAFs), myeloid-derived suppressor cells (MDSCs), cancer-associated mesenchymal stem cells (MSCs), regulatory T cells (Tregs), along with other cell types (**Figure 2**).

### 3.1 Dendritic cells

As the most important antigen-presenting cell subtype to T lymphocytes responsible for initiating immune responses, DCs can connect innate immune system with adaptive immune system. However, CSCs alter DC phenotypes and render them immunosuppressive, thus impeding the activation of T cells and anti-tumor immune responses. In colon cancer, a previous study indicated that DCs-secreted CXCL1 enhanced cell migration, EMT and cancer metastatic ability as well as increasing expression of oncogene (PTHLH, TYRP1, FOXO1, TCF4 and ZNF880), CSC-related transcriptional factors (Nanog, Oct4 and Sox2) and miR-105 (49).

### 3.2 Tumor-associated macrophages

TAMs are divided into M1 pro-inflammatory phenotype and M2 pro-tumorigenic phenotype. In gastrointestinal cancer, CSCs promote TAMs recruitment by secreting CC chemokine family members (CCL2, CCL5), CXC chemokine family members (CXCL5, CXCL12), and soluble proteins like colony-stimulating factor1 (CSF1), MIC-1, LOX and VEGF (50). CSCs can also stimulate TAMs polarization into M2 phenotype by secreting IL-13, IL-34, CSF2, TGF-β, osteoactivin and exosomes (50). In turn, as the predominant immune cell type within CSCs niche, infiltrated TAMs can enhance CSCs properties and facilize CSCs maintenance through the secretion of various cytokines and soluble molecules including CCL8, CCL17, CCL22, IL-6, IL-18, TGF-β, hCAP-18/LL-37, S100A9, MFG-E8 and extracellular vesicles (50). For example, in hepatocellular carcinoma, TGF-β produced by TAMs induced NF-κB, AKT and STAT3 signaling pathways in CSCs, thus enhancing HCC stemness and epithelial to mesenchymal transition (51). Besides, TAMs-secreted IL-6 promoted CD44^+^ HCC CSCs expansion *via* STAT3 (52). In colorectal cancer, transforming growth factor-β (TGF-β) can promote stem cell-like properties (53). In pancreatic cancer, CSCs were detected to overexpress TGF-β superfamily members Nodal and Activin, which drive self-renewal and *in vivo* tumorigenicity (54). TAMs produce leucine leucine-37 (LL-37) to respond to CSCs-secreted TGF-β1, Nodal and Activin A, leading to PDAC progression and metastasis, which can be inhibited by targeting the LL-37 receptors FPR2 and P2X7R (55).

### 3.3 Cancer-associated fibroblasts

CAFs regulate CSCs properties to support tumor growth through specific factors and pathways in gastrointestinal cancers such as gastric (56), colorectal (57, 58) and liver cancers (59, 60). Reciprocally, CSCs will enhance the proliferation and maintain the immunosuppressive phenotype of CAFs (61). Recently, it has been reported that cancer-associated fibroblasts (CAFs) exert an effect on educating MDSCs and thus promoting stemness of intrahepatic cholangiocarcinoma through the LTB4-BLT2 axis (62). CAFs can enhance the self-renewal and the frequency of PDAC CSCs *via* integrin-FAK signaling (63). NRG1 secreted by CAFs promotes the self-renewal of gastric CSCs through the activation of NF-κB signaling (64). Furthermore, a recent study uncovered that CAFs can promote cancer stemness through the osteopontin/secreted phosphoprotein 1-CD44 axis in pancreatic cancer (65). CAFs-secreted hepatocyte growth factor (HGF) and IL-6 were reported to enhance the stemness of CD24^+^ HCC cells through STAT3 signaling (59). CAFs can sustain the stemness of gastric CSCs *via* acting on TGF-β signaling (56). HGF secreted by CAFs can regulate CSCs stemness of hepatocellular carcinoma and colorectal cancer by activating c-Met/FRA1/HEY1 signaling, Wnt/β-catenin and PI3K signaling, respectively (60, 66).

### 3.4 Myeloid-derived suppressor cells

MDSCs are categorized into monocytic MDSCs (M-MDSCs) and polymorphonuclear MDSCs (PMN-MDSCs). They are essential cellular components mediating immunosuppression. Substantial evidence has revealed MDSCs are closely linked with cancer stemness maintenance (67, 68). In esophageal squamous cell carcinoma (ESCC), MDSCs can activate NEDD9 to enhance cancer stemness with the involvement of Notch signaling (69). By the activation of STAT3, MDSCs can enlarge ALDH1(Bright) CSCs population derived from pancreatic cancer (70). Additionally, a previous study showed MDSCs can confer breast cancer cells stem-like properties (71).

### 3.5 Cancer-associated mesenchymal stem cells

MSCs produce diverse cytokines to facilize CSCs restoration in the TME (72, 73). They can support the reacquisition and maintenance of gastric CSCs by the activations of the WNT and TGF-β signaling pathways (72). In addition, MSCs derived from gastric cancer can secrete IL-6 and IL-8 to trigger TAMs polarization into M2 macrophages through the JAK2/STAT3 signaling pathway (74).

### 3.6 Regulatory T cells

As a subtype of CD4^+^ T cells, Treg cells are capable of promoting cancer progression by negatively regulating immune response. CSCs recruit Treg cells into the tumor microenvironment through releasing relevant factors such as CCL1, CCL2 and CCL5. In turn, Treg cells contribute to the formation of immunosuppressive tumor environment. In one study of CRC, it has been found that IL-17-expressing Treg cells can induce cancer-initiating cells (75).

It is revealed that CSCs modulate immune cells behaviors by secreting various immunosuppressive cytokines and soluble factors like IL-4, IL-6, IL-8, IL-10, IL-13, and TGF-β (76–78). IL-4 was detected to be overexpressed on CICs isolated from CRC, representing one important actor that affects T lymphocytes-mediated anti-tumor activity (30). IL-4 signaling blockade can be regarded as a useful target of CRC CICs. Similarly, it was demonstrated that disrupting IL-8/CXCR1 signaling involved in pancreatic CSCs stemness would lead to the reduction of CSCs population (76). Furthermore, TGF-β stimulates the differentiation of regulatory T cells (Treg) and promotes fibrosis to make negative effects on effector T-cell proliferation and infiltration (78, 79). In addition to CSCs, TGF-β can also be produced by TAMs, CAFs, lymphocytes and mesenchymal stem cells (MSCs) (80–82). Collectively, this compelling evidence implicates the complex crosstalk between GCSCs and cells in the TME plays a vital part in the regulation of GCSCs.

## 4 Immunotherapeutic approaches against CSCs in gastrointestinal malignancies

**Figure 3:**
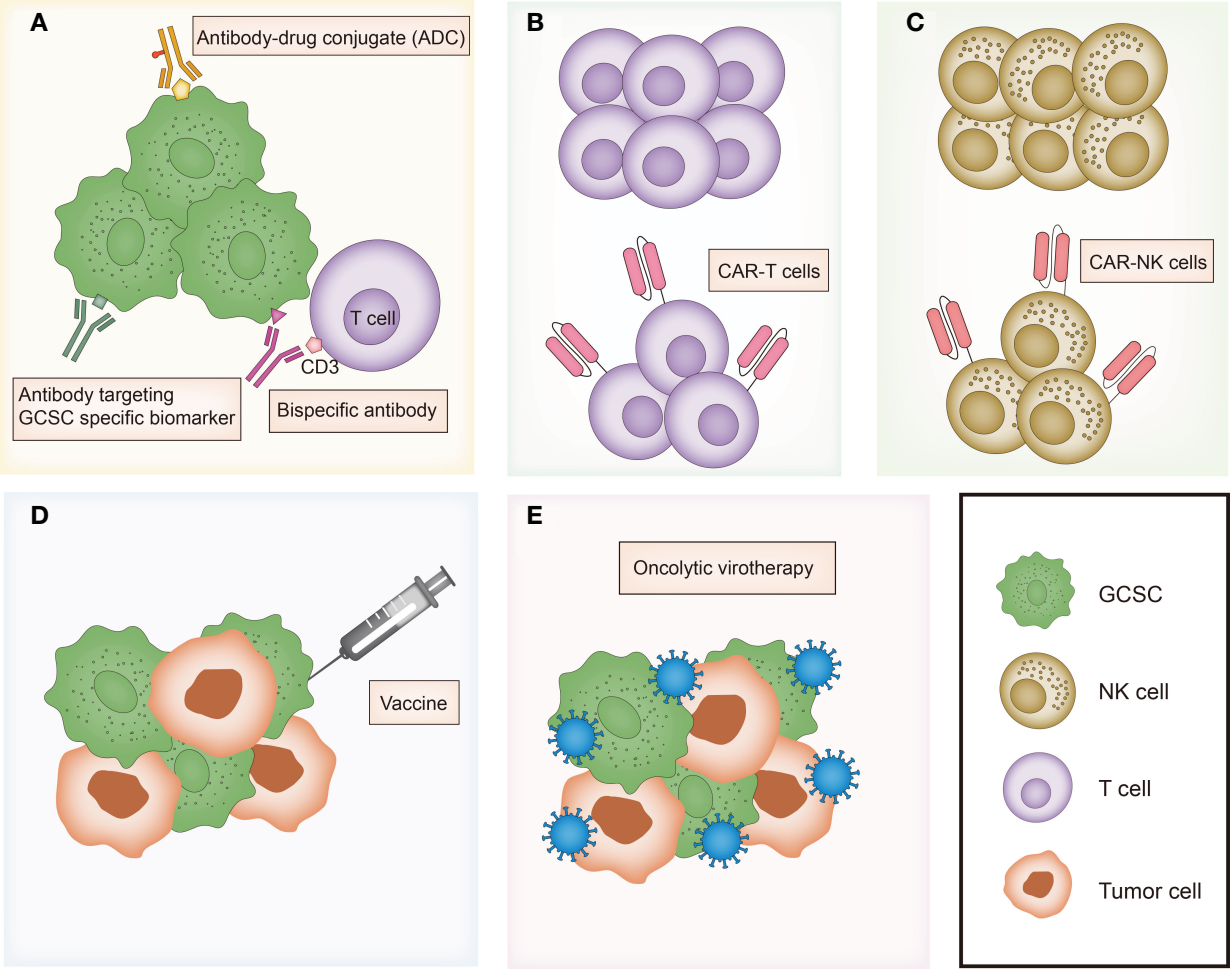
Overview of major immunotherapies targeting gastrointestinal cancer stem cells. **(A)** Antibody-mediated immunotherapies including antibody targeting GCSC specific biomarker, bispecific antibody and antibody-drug conjugate (ADC). **(B)** T cell-based immunotherapy. **(C)** NK cell-based immunotherapy. **(D)** Vaccines targeting GCSC. **(E)** Oncolytic virotherapy targeting GCSC. GCSC, gastrointestinal cancer stem cell; CAR, chimeric antigen receptor.

In the last recent decades, cancer immunotherapy has obtained enormous attention. Considering that GCSCs play a critical role in drug resistance and therapeutic failure, immunotherapeutic approaches that target GCSCs can be a promising research field. Diverse anti-GCSC immunotherapeutic approaches have been designed and developed including antibody-mediated immunotherapy, immune cell-based immunotherapy, vaccines and oncolytic virotherapy (**Figure 3**). Preclinical studies and clinical trials associated with multiple immunotherapies targeting GCSCs have been listed in **Tables 1**, **2**, respectively.

**Table 1:** Preclinical studies of immune-mediated therapies targeting GCSCs.

Immunological strategy		Cancer types	Effects	References
**Antibody**	Anti-CD44 monoclonal antibody	Recurrent pancreatic ductal adenocarcinoma (PDAC)	Reduced PDAC CSCs in mice carrying PDAC-derived xenografts previously treated with gemcitabine.	(83)
	Anti-CD133 monoclonal antibody CMab-43	Colon cancer	Inhibited tumor development in xenograft mouse model.	(84)
	Asymmetric BiAb consisting of monomer of chimeric AC133 (CD133 mAb) and single chain of humanized OKT3	Colorectal cancer	Inhibited CD133^high^ colorectal cancer cells and tumor growth by arming activated T cells *in vitro* and *in vivo*.	(85)
**Antibody-drug conjugate (ADC)**	Anti-CD133-drug conjugate (AC133-vcMMAF)	Hepatocellular cancer, gastric cancer	Inhibited CD133^+^ CSCs growth and delayed tumor growth in mouse models.	(86)
	Anti-LGR5-drug conjugate	Colon cancer	Suppressed tumorigenesis of CSCs and extended survival in mouse models.	(87)
	Anti-5T4-drug conjugate (H6-DM4)	Colorectal cancer	Eliminated colorectal CICs *in vitro* and *in vivo*.	(88)
	Anti-CD24 antibody G7mAb conjugated with doxorubicin (G7mAb-DOX)	Hepatocellular carcinoma	Inhibited tumor growth in hepatocellular carcinoma-bearing mice.	(89)
	hG7-BM3-VcMMAE conjugate	Hepatocellular carcinoma	Inhibited the proliferation of tumor cells and the growth of tumor *in vivo*.	(90)
**Immune cell-based therapy**	CTLs specific for OR7C1 peptide	Colon cancer	Inhibited tumor growth in a CTL adoptive transfer mouse model.	(91)
	CTLs specific for ASB4	Colorectal cancer	Prevented tumor growth in a mouse model.	(92)
	Anti-EpCAM CAR-T	Colorectal cancer with peritoneal metastasis	Delayed tumor progression in mouse models.	(93)
	CAR-NK-92 cells against EpCAM^+^ cancer cells plus regorafenib	Colorectal cancer	Enhanced tumor growth suppression in a mouse model.	(94)
	NK cells activated by CD133	Gastric cancer	Killed gastric CSCs in an NKG2D-dependent manner.	(95)
	CSCs antigen loaded DC-CIK cells	Liver cancer	Suppressed CSCs growth *in vivo* and inhibited tumor growth in mouse models.	(96)
	CIK cells bound with anti-CD3/anti-CD133 BsAb (BsAb-CIK)	Pancreatic cancer, hepatic cancer	Inhibited CD133 ^high^ CSCs and tumor growth *in vitro* and *in vivo*.	(97)
**Vaccine**	DC vaccine pulsed with CSC-derived DRibbles	Colorectal carcinoma	Suppressed cancer growth and prolonged the survival of mice.	(98)
	DC vaccine loaded with Panc-1 CSCs lysates	Pancreatic cancer	Elicited antitumor immune killing *in vitro*.	(99)
**Oncolytic virotherapy**	CD133-targeted oncolytic adenovirus (AdML-TYML)	Colorectal cancer	Inhibited tumor formation in mouse models.	(100)
	AdNuPARmE1A	Pancreatic cancer	Reduced CSCs *in vitro* and inhibited tumor formation in mouse models.	(101)
	Ad/TRAIL-E1	Esophageal cancer	Killed CSCs and suppressed tumor growth in mouse models.	(102)
	OBP-301	Gastric cancer	Killed quiescent CD133^+^ stem-like cells.	(103)
	GP73-regulated oncolytic adenovirus GD55	Liver cancer	Killed liver CSCs *in vitro* and *in vivo*.	(104)
	Cancer-favoring oncolytic vaccinia virus (CVV)	Colon cancer	Suppressed stem-like cells of colon cancer.	(105)

**Table 2:** Summary of clinical trials using immunological strategies targeting GCSCs.

Immunological strategy		Target	Tumor type	NCT Number	Phase	Status
**Antibody**	RO5429083 (RG7356)	CD44	CD44-expressing malignant solid tumors	NCT01358903	I	Completed
	Catumaxomab (Removab)	EpCAM	Gastric adenocarcinomas with peritoneal carcinomatosis	NCT01504256	II	Completed
	Catumaxomab (Removab)	EpCAM	EpCAM positive tumor (e.g., gastric, colon), malignant ascites	NCT00836654	II/III	Completed
	Catumaxomab (Removab)	EpCAM	Malignant ascites due to epithelial cancer	NCT00822809	III	Completed
	Catumaxomab (Removab)	EpCAM	Gastric cancer, gastric adenocarcinoma	NCT00464893	II	Completed
	MT110: anti-EpCAM and anti-CD3 bispecific T-cell engager (BiTE)	EpCAM	Solid tumors	NCT00635596	I	Completed
	BNC101: anti-LGR5 humanized mAb	LGR5	Colorectal cancer	NCT02726334	I	Terminated
	MCLA-158: bispecific antibody targeting EGFR and LGR5	LGR5	Advanced/metastatic solid tumors, colorectal cancer	NCT03526835	I	Unknown
**CAR-T**		EpCAM	Advanced solid tumor	NCT04151186	n.a.	Unknown
		EpCAM	Advanced gastric cancer with peritoneal metastasis	NCT03563326	I	Recruiting
		EpCAM	Stomach cancer	NCT02725125	n.a.	Unknown
		EpCAM	Liver cancer	NCT02729493	n.a.	Unknown
		EpCAM	Colon cancer, esophageal carcinoma, pancreatic cancer	NCT03013712	I/II	Unknown
		EpCAM	Advanced hepatocellular carcinoma, colorectal cancer, gastric cancer, pancreatic cancer	NCT05028933	I	Recruiting
		EpCAM	Advanced solid tumors	NCT02915445	I	Recruiting
		CD133	Liver cancer, pancreatic cancer, colorectal cancer, gastric cancer	NCT02541370	I/II	Completed
**CAR-NK**		MUC1	MUC1 positive relapsed or refractory solid tumor	NCT02839954	I/II	Unknown
**Vaccine**	DC vaccine loaded with CSC		Hepatocellular cancer	NCT02089919	I/II	Completed
	DC vaccine loaded with CSC		Colorectal cancer	NCT02176746	I/II	Completed
	DC vaccine loaded with CSC		Pancreatic cancer	NCT02074046	I/II	Completed
**Oncolytic virotherapy**	Enadenotucirev		Colorectal cancer	NCT02636036	I	Completed

### 4.1 GCSCs biomarkers

Various biomarkers have been applied for CSCs-targeting therapeutic strategies. Representative biomarkers for CSCs identification in gastrointestinal malignancies are summarized in **Figure 1**.

CD133 (human prominin-1), a kind of glycoprotein with five transmembrane regions, has been identified as a valid CSC marker in gastrointestinal cancers including gastric, liver, colorectal, pancreatic cancers (106–109) and other cancers like lung (110), brain (10), and prostate cancers (16). In hepatocellular carcinoma, CD133 was reported to facilitate CSC-like features by stabilizing EGFR-AKT signaling (111).

CD24, a mucin-like cell surface glycoprotein, has been regarded as a “don’t eat me” signal (112). By binding to inhibitory receptor sialic-acid-binding Ig-like lectin 10 (Siglec-10), CD24 overexpressed on liver CSCs prevents macrophage phagocytosis. J Ke et al. (113) demonstrated that CD24^+^ subpopulation of colon cancer cell lines showed CSCs properties like self-renewal capacity and tumorigenicity ability compared to CD24^-^ cancer cells.

The epithelial cell adhesion molecule (EpCAM, CD326) is a multi-functional transmembrane glycoprotein overexpressed in normal epithelial cell and epithelial carcinomas such as cancers of pancreas, colon, stomach, lung, ovarian and so on (114, 115). Functionally, it plays a significant role in modulating intercellular cell-adhesion, cell signaling, proliferation, epithelial-to-mesenchymal transition and stemness of cancer cells (114, 116, 117), known to be a marker for CSCs in liver cancer and colorectal cancer (118, 119). In a recent study, researchers identified a high-risk tumor subtype of hepatocellular carcinoma using intratumoral EpCAM^+^ cancer stem cell (120).

In addition to well-known identified CSC biomarkers, other novel and potential GCSC biomarkers also have been found. Doublecortin-like kinase1 (DCLK1) is mentioned as a CSC marker of gastrointestinal cancers such as colon cancer (121, 122), pancreatic cancer (123) and liver cancer (124). One recent study stated that DCLK1 can also express on cholangiocarcinoma (CCA) CSC subpopulations of iCCACD133^+^ and pCCALGR5^+^ (125). Glypican-3 (GPC3) was reported to mediate the CSC properties like self-renewal, cell cycle progression, and tumor formation *via* autophagy induction in hepatocellular carcinoma, implicating it as a novel liver CSC marker (126). Moreover, heat shock protein DNAJB8 has been found to be a novel CSC/CIC antigen in CRC (127). As the melanoma antigen gene (MAGE) family number, MAGE-A9 exhibits a higher level of expression in EpCAM^+^ HCC cells, contributing to the stemness of hepatocellular carcinoma (128).

Notably, CSCs accounting for only a small proportion of cancer tissues have been shown to share several similar biomarker profiles with normal stem cells, indicating that therapeutic agents targeting biomarkers may kill healthy normal tissue stem cells instead of CSCs, which will bring serious problems like drug toxicity and tumor recurrence during anticancer treatment (129–131). For example, LGR5 expression is not restricted to gastric, CRC CSCs, but it can also be expressed in healthy intestine stem cells. Thus, identifying unique markers and specific cell surface antigens of CSCs compared to non-CSCs is of utmost importance to CSC-directed immunotherapies. Multiple cancer testis antigens were preferentially expressed in CSCs rather than non-CSCs (132), suggesting potential antigens for immunotherapy targets.

### 4.2 Antibody-mediated immunotherapies

With advances in understanding the key role GCSCs plays in gastrointestinal cancers initiation and development, there is a growing concern in targeting GCSCs. In order to diminish and deplete the GCSCs, antibody-mediated treatment modalities have been developed and tested in diverse studies. For example, a first-in-human, phase I, two-arm clinical trial (NCT01358903) involving patients with advanced-stage CD44^+^ solid tumors showed RG7356 (anti-CD44 monoclonal antibody) can be well tolerated in spite of its modest clinical efficacy (133). Further, nanoparticles can be taken as the drug carrier to enhance the efficacy of antibody immunotherapy. The anti-CD133 antibody-conjugated SN-38-loaded nanoparticles named CD133Ab-NPs-SN-38 has been designed to target CD133^+^ cells in colorectal cancer, and the results indicated that it could eliminate and suppress cancer growth and recurrence in an HCT116 xenograft model (134).

The combined use of different biomarkers has demonstrated enhanced therapeutic effects. For instance, the bispecific and trifunctional antibody catumaxomab, which can simultaneously bind to T cells, macrophages, DC, and NK-cells with two antigen-binding sites including EpCAM and CD3, has been used to treat CD133^+^/EpCAM^+^ epithelial cancer patients suffering from malignant ascites (135–137). A clinical study (NCT00836654) was conducted in EpCAM^+^ cancer patients suffering from malignant ascites, demonstrating that catumaxomab treatment slowed deterioration in quality of life (QoL) for patients to achieve a prolonged survival period (138). In addition, a phase II study (NCT01504256) indicated the efficacy of catumaxomab in gastric adenocarcinoma patients with peritoneal carcinomatosis, and meanwhile, severe side effects regarding catumaxomab were revealed (139).

Apart from the common antibodies against GCSCs biomarkers, antibody-drug conjugate (ADC)-directed immunotherapy has been developed as a favorable therapeutic approach. Comprised of antibody with exquisite specificity and cytotoxic drug with cell-killing efficiency, ADC is an innovative antitumor weapon to fight against GCSCs, as evidenced by the accumulating studies. L M Smith et al. used anti-CD133 ADC, AC133-vcMMAF to eradicate CD133^+^ tumor cells including CSCs in hepatocellular and gastric cancers (86). In addition, LGR5-targeted ADCs were effective in the eradication of LGR5^+^ gastrointestinal cancers and the suppression of tumor progression (87, 140). Trophoblast glycoprotein 5T4 expression exhibits an increased level in colorectal CICs compared with non-CICs, and an anti-5T4 antibody in combination with potent microtubule inhibitor DM4 showed powerful efficacy against colorectal CICs *in vitro* and *in vivo* (88). Further, in preclinical models of hepatocellular carcinoma, anti-CD24 ADCs including CD24 antibody-conjugated doxorubicin G7mAb-DOX and the hG7-BM3-VcMMAE conjugates showed antitumor activities *in vivo* (89, 90).

### 4.3 Immune cell-based immunotherapies

Immune cell-based immunotherapies such as T cell-based immunotherapy, NK cell-based immunotherapy and cytokine-induced killer (CIK) cell-based immunotherapy have enormous potential for the treatment of gastrointestinal cancers.

#### 4.3.1 T cell-based immunotherapy

CSC-primed T cells targeting CSCs strictly depend on the normal and intact antigen processing and presenting machinery, and the absence of antigens may lead to the failure of targeting CSCs. The olfactory receptor family 7 subfamily C member 1 (OR7C1) was identified as a potential functional marker of colon CICs and its overexpression correlates with poor prognosis of CRC patients (91). A cytotoxic T lymphocytes (CTL) adoptive transfer mouse model applying CTLs specific for OR7C1 peptide was constructed to exert specific cytotoxicity targeting colon CICs. Another study reported that ankyrin repeat and SOCS box protein 4 (ASB4) can elicit CD8^+^ cytotoxic T cell responses against colorectal CSCs but not non-CSCs because of its specific and unique expression in CSCs (92). *In vivo* models, adoptive transfer of CTLs specific for ASB4 significantly prevented tumor development.

The chimeric antigen receptor-modified T (CAR-T) cell immunotherapy can specially recognize and kill cancer cells using engineered T lymphocytes with the expression of chimeric antigen receptor (CAR) on their surface. To date, CAR-T immunotherapies based on specific biomarkers have been explored in a variety of preclinical studies and clinical trials. Wei Xia Ang et al. established peritoneal dissemination mouse models of human colorectal cancer in immunodeficient NSG mice, demonstrating that anti-EpCAM CAR-T immunotherapy can suppress and delay the development of peritoneal tumors (93). Another preclinical study of the adoptive transfer of EpCAM CAR-T cells indicated that the growth and formation of colon cancers were delayed *in vivo* mouse model, without serious adverse effects (141). Clinical trials using EpCAM targeted CAR-T therapy have been conducted in a broad spectrum of gastrointestinal cancers, as depicted in **Table 2**. A phase I trial (NCT02541370) showed the clinical efficacy and controllable side effects of autologous CAR-T cell directed CD133 termed CART-133 to treat patients with CD133^+^ advanced metastasis malignancies, and it seemed that CART-133 cells exhibited the “on-target, off-tumor” effect on patients with bile duct stenosis for the reason that it can target CD133 antigen exposed on the bile duct endothelium (142). Kai-chao Feng et al. reported EGFR-specific and CD133-specific CAR-T sequential treatment defined as CAR-T cocktail immunotherapy in a case of advanced cholangiocarcinoma, in which the patient achieved certain clinical response, but the accompanied toxicities should not be ignored (143). In fact, using CAR-T therapy can offer optimistic efficacy for cancer treatment, but we may face much difficulties owing to PD-L1 existence and T cell exhaustion (144, 145).

#### 4.3.2 NK cell-based immunotherapy

NK cells are able to selectively kill GCSCs through modulating the expression of activating and inhibitory receptors. For example, it has been observed that NK cells can kill CD133^+^ CSCs of colon and gastric cancers (95, 146). *In vivo* adoptive NK cells transfer model of pancreatic cancer, researchers found NK cells can target CSCs and attack them, leading to the reduction of CSC population and the delayed tumor progression (147). Moreover, Erik Ames and colleagues (148) indicated that the combination of NK cell adoptive immunotherapy with local radiation therapy (RT) had synergistic effects on CSCs elimination and tumor growth suppression *in vitro* and *in vivo*. RT can sensitize CSCs derived from solid tumors like pancreatic cancer to NK cells attack by promoting the expression of NK cell ligands on CSCs. This study provided evidence for adoptive cell therapy in conjunction with other cytotoxic standard therapies for complete tumor eradication and long-term clinical benefits in multiple solid tumors.

Compared with CAR-T immunotherapy, CAR-NK cell therapy can reduce on-target toxicity (149, 150). Researchers also constructed EpCAM targeted second-generation CAR and transduced it into NK-92 cells using lentiviral vectors, and they indicated CAR-NK-92 cells can specially kill EpCAM^+^ colorectal cancer cells and combination with regorafenib can greatly reduce tumor growth in NOD/SCID mice with human colorectal xenograft models (94). A clinical trial (NCT02839954) concerning the immunotherapy of anti-MUC1 CAR-pNK cells has been conducted in patients with MUC1^+^ solid tumors.

#### 4.3.3 CIK cell-based immunotherapy

Furthermore, cytokine-induced killer (CIK) cells loaded with the anti-CD3/anti-CD133 BsAb (BsAb-CIK) can selectively eliminate CD133^high^ cancer cells of SW1990 pancreatic cancer cell line and Hep3B hepatic cancer cell line (97). Moreover, BsAb-CIK treatment in a mouse model led to the suppression of CD133^high^ tumor growth. A previous study indicated that the combined strategy of DCs loaded with liver cancer stem cells (LCSC) antigens and CIK cells exhibited a significant inhibitory effect on HCC tumor growth and LCSC growth (96).

### 4.4 Vaccines

Vaccination based on CSC lysates exhibit efficacious antitumor ability in preclinical and clinical studies. Mei Guo et al. suggested that colorectal CSCs lysates-based vaccine served as an effective and safe anti-colorectal cancer vaccine (151). In this study, molecule mucin1 (MUC1), a tumor associated antigen overexpressed in colorectal CSCs in comparison with WT SW620 cells, was reported to be required for colorectal CSCs-based vaccine to exert anti-tumor immunity (151, 152). Furthermore, in a clinical trial carried out among 90 patients with pancreatic adenocarcinoma in 2014, pancreatic CSC vaccine was preliminarily proved to be safe and effective (153).

Another strategy, dendritic cells (DCs) based anti-tumor vaccination is capable of inducing cytotoxic T lymphocytes activities against CSCs. Several DC-based vaccines have been devised to cure patients with gastrointestinal cancers. Different components including RNAs extracted from cancer cells, tumor lysates, and tumor-derived peptides can be used as antigens to induce dendritic cells activities. Vahid Bagheri et al. utilized DCs pulsed with mRNA of gastric CSCs isolated from patients to prime *in vitro* effective T cell-mediated immune responses (154). Similarly, in 2010, Jian-cong Sun et al. developed a DC-based vaccine loaded with CD133^+^ HCC cells total RNA, which can generate antigen-specific cytotoxic T lymphocytes response targeting HCC CSCs (155). Compared with RNA-loaded DCs, DCs pulsed with lysates of colon cancer stem cells (CCSCs) isolated from CD44^+^ CT-26 colon cancer cells can evoke stronger anti-tumor immune responses against CCSCs (156). Apart from that, a recent study applying a D5 melanoma model indicated ALDH 1A1+1A3 dual peptides-DC vaccine can significantly reduce ALDH^high^ CSCs and enhance anti-PD-L1 efficacy (157).

Recently, tumor-cell derived autophagosomes (Dribbles), which can serve as a kind of DC-pulsed antigen due to the high immunogenicity, have been used for DC-based vaccination in diverse cancers such as oral squamous cell carcinoma (158), head and neck cancer (159), colorectal carcinoma (98) and hepatocellular carcinoma (160). Of interest, Changhao Fu et al. obtained defective ribosomal products-containing autophagosome-rich blebs from CD44^+^ colon CSCs and generated DC vaccines pulsed with CCSC-derived Dribbles, demonstrating the efficient cytotoxic T-cells immune responses and tumor growth inhibition in mice model of colorectal carcinoma (98).

### 4.5 Oncolytic virotherapy

A growing number of evidence has elucidated that oncolytic viruses (OVs) can potentially kill both differentiated cancer cells within tumor bulks and CSCs through multiple ways (161). Several preclinical studies indicated that GCSCs resistant to conventional treatment modalities can be efficiently eradicated by oncolytic virotherapy-mediated killing, which have been indicated in **Table 1**.

As mentioned above, promising preclinical experiments or clinical trials administering immunotherapy have been conducted and reported to target GCSCs. Indeed, safe and successful clinical use of drugs requires further studies and investigations, and it is equally important to identify specific GCSCs biomarkers. Moreover, eliminating GCSCs can affect tumor growth, but to achieve complete elimination of tumor mass, it is a feasible strategy that combines anti-GCSCs immunotherapy with other anti-tumor therapies such as chemotherapy and radiotherapy.

## 5 Conclusion

Taken together, GCSCs can evade immune attack through the downregulation of MHC-I and natural killer ligands and the upregulation of immune checkpoint molecules. And the immunosuppressive niche provides a supportive room for them to survive and maintain properties, contributing to the development of malignant cancers. Current efforts aimed at developing GCSCs-targeted immunotherapies in gastrointestinal cancers have shown preclinical and clinical success, but it is always a thought-provoking question to selectively target GCSCs efficiently and accurately. In addition, it is a promising and effective method to combine GCSCs-directed immunotherapy with conventional therapies like chemotherapy and radiotherapy. Despite these advances, a tremendous amount of efforts need to be put into the development of efficacious immunotherapies targeting GCSCs.

## Author contributions

JA wrote the manuscript and designed the figures, FL and XH designed the manuscript. All authors contributed to the article and approved the submitted version.
